# Fully Automated Spectrometric Protocols for Determination of Antioxidant Activity: Advantages and Disadvantages

**DOI:** 10.3390/molecules15128618

**Published:** 2010-11-29

**Authors:** Jiri Sochor, Marketa Ryvolova, Olga Krystofova, Petr Salas, Jaromir Hubalek, Vojtech Adam, Libuse Trnkova, Ladislav Havel, Miroslava Beklova, Josef Zehnalek, Ivo Provaznik, Rene Kizek

**Affiliations:** 1 Department of Breeding and Propagation of Horticultural Plants, Faculty of Horticulture, Mendel university in Brno, Valticka 337, CZ-691 44 Lednice, Czech Republic; 2 Department of Chemistry and Biochemistry, Faculty of Agronomy, Mendel University in Brno, Zemedelska 1, CZ-613 00 Brno, Czech Republic; 3 Department of Microelectronics, Faculty of Electrical Engineering and Communication, Brno University of Technology, Udolni 53, CZ-602 00 Brno, Czech Republic; 4 Research Centre for Environmental Chemistry and Ecotoxicology, Faculty of Science, Masaryk University, Kotlarska 2, CZ-611 37 Brno, Czech Republic; 5 Department of Plant Biology, Faculty of Agronomy, Mendel University in Brno, Zemedelska 1, CZ-613 00 Brno, Czech Republic; 6 Department of Veterinary Ecology and Environmental Protection, Faculty of Veterinary Hygiene and Ecology, University of Veterinary and Pharmaceutical Sciences, Palackeho 1-3, CZ-612 42 Brno, Czech Republic; 7 Department of Biomedical Engineering, Faculty of Electrical Engineering and Communication, Brno University of Technology, Kolejni 4, CZ-612 00 Brno, Czech Republic; 8 Department of Natural Drugs, Faculty of Pharmacy University of Veterinary and Pharmaceutical Sciences, Palackeho 1-3, CZ-612 42 Brno, Czech Republic

**Keywords:** antioxidant activity, spectrometry, trolox, gallic acid

## Abstract

The aim of this study was to describe behaviour, kinetics, time courses and limitations of the six different fully automated spectrometric methods - DPPH, TEAC, FRAP, DMPD, Free Radicals and Blue CrO_5_. Absorption curves were measured and absorbance maxima were found. All methods were calibrated using the standard compounds Trolox^®^ and/or gallic acid. Calibration curves were determined (relative standard deviation was within the range from 1.5 to 2.5 %). The obtained characteristics were compared and discussed. Moreover, the data obtained were applied to optimize and to automate all mentioned protocols. Automatic analyzer allowed us to analyse simultaneously larger set of samples, to decrease the measurement time, to eliminate the errors and to provide data of higher quality in comparison to manual analysis. The total time of analysis for one sample was decreased to 10 min for all six methods. In contrary, the total time of manual spectrometric determination was approximately 120 min. The obtained data provided good correlations between studied methods (R = 0.97 – 0.99).

## 1. Introduction

Free radicals (FRs) are naturally formed in a wide range of biological as well as chemical systems. They are chemical stable atoms and molecules, which have one (or rarely more) free electron/electrons in the electron envelope [1,2,3]. Almost all biomolecules, but mainly biomembranes, proteins and nucleic acids, may be attacked by reactive free radicals. Free radicals are responsible for many pathological processes, or they can be generated as the result of the pathological stage and cause important secondary damage to biological systems and cells [1,4,5,6,7]. Connections between free radicals and some serious diseases, including Parkinson´s and Alzheimer´s disease, atherosclerosis, heart attacks, and chronic fatigue syndrome, have been demonstrated. However, short-term oxidative stress (OS), the unbalance between the formation and scavenging of the reactive oxygen species, may be important in the prevention of aging due to the triggering the process known as mitohormesis [6,8,9,10,11,12,13]. On the average, 65 – 70 % of the population is excessively impacted by oxidative stress caused by FRs. Therefore, OS monitoring is an important part of reasonable health prevention [4,8,9,14,15,16,17,18].

The protective system of the organisms is based on the activity of specific enzymes (especially superoxide dismutase, glutathione peroxidase, catalase, glutathione reductase) as well as non-enzymatic compounds with antioxidant activity (α-tocopherol, L-ascorbic acid, glutathione, coenzyme Q10, flavonoids, albumin and other still unidentified molecules) called antioxidants [1,8,10,11,12,15,19,20,21,22]. This intricately linked system provides a hydrogen radical, which is able to react with the reactive free radical forming a neutral compound [14,23]. The antioxidant activity is one of the ways how to define the ability of an organism to protect itself against free radicals. It is defined as an ability of the compound (or mixture of compounds) to inhibit oxidative reaction of various biomolecules (e.g. prevent the peroxidation of lipids). Methods of the antioxidant activity determination are usually based on the direct reaction of the studied molecule with radicals (scavenging) or on the reaction with transition metals [3,14,17,24]. 

Determination of the antioxidant activity is one of the ways how to biologically and nutritionally evaluate the quality of the fruit. It has been proved that antioxidant activity depends on the type of phenolics present in the fruit, as some phenolic compounds exhibit higher antioxidant activity than others [7,9,19,20,21,22,25,26,27,28,29]. It is assumed that the ability of plant polyphenols to scavenge reactive oxygen radicals participates in the protective mechanism of plants. Due to the chemical diversity of antioxidants present in fruit, their strictly defined content is unavailable. In spite of the fact that total amount of antioxidants in various fruit types need not to represent the total antioxidant capacity [2,4,5,7,9,19,20,21,22,25,26,28,29,30,31,32,33,34,35], almost all phenolic compounds in fruits demonstrate some antioxidant activity [1,2,3,4,7,9,10,11,12,13,16,17,18,19,20,21,22,23,24,25,26,27,29,32,33,34,35,36,37,38,39,40]. However, detection of therapeutically active components in a biological matrix is a very complex procedure, and their determination differs in individual studies [41,42,43,44,45].

This study is focused on the optimization and precise description of six photometric protocols for automated detection of the antioxidant activity of biological samples (to express the antioxidant activity of the blood serum, various fruits and food products). These methods are mutually compared and their advantages and disadvantages, including usage of manual and automated analyzer, are discussed.

## 2. Results and Discussion

In the field of chemical analyses and biological evaluation of the antioxidant characteristics several methods enabling determination of the antioxidant activity have been suggested and optimized [3,14,15,17,39]. These methods are principally different and their modifications are still progressively developing. Their importance lies in the characterization of the antioxidant activity under conditions similar to physiological conditions, however, the majority of these methods are not optimized for fully automated analysis [28]. In the first part of our study, protocols, in which individual methods are characterized in detail including preparation of reagents, conditions and processes of measurements as well as calculating of the data obtained for determination of antioxidant activity for individual tests, are described.

### 2.1. Protocols

#### 2.1.1. Determination of antioxidant activity by the DPPH^•^ test

The DPPH^•^ test is based on the ability of the stable 2,2-diphenyl-1-picrylhydrazyl free radical to react with hydrogen donors. The DPPH^•^ radical displays an intense UV-VIS absorption spectrum. In this test, a solution of radical is decolourized after reduction with an antioxidant (AH) or a radical (R^•^) in accordance with the following scheme: DPPH^•^ + AH → DPPH^•^-H + A^•^, DPPH^•^ + R^•^ → DPPH^•^-R [39]. This method is very simple and also quick for manual analysis. 

*Reagent preparation:* 0.95 mmol·L^-1^ solution of radical DPPH^•^ (m = 0.00374 g/100 mL). First, this amount is dissolved in 50 mL of DMSO and after dissolution made up to a volume of 100 mL with ACS water. The solution can be used for 7 days when stored at 4 °C and in the dark. 

*Measurement procedure for an automated analyzer:* A 200 µL volume of reagent is incubated with 20 µL of sample (gallic acid, Trolox^®^). Absorbance is measured after 1,520 seconds at λ = 510 nm. To calculate the antioxidant activity, the values determined before decrease of the absorbance (224^th^ second of measurement – A_224_) and the last measurement value (1520^th^ second of measurement – A_1520_) are used. Resulting value is calculated in accordance with the following formula: A = A_1520_-A_224_. 

#### 2.1.2. Determination of antioxidant activity by the ABTS test

The ABTS radical method is one of the most used assays for the determination of the concentration of free radicals. It is based on the neutralization of a radical-cation arising from the one-electron oxidation of the synthetic chromophore 2,2‘-azino-bis(3-ethylbenzothiazoline-6-sulfonic acid) (ABTS^•^): ABTS^•^ – e- ABTS^•+^. This reaction is monitored spectrophotometrically by the change of the absorption spectrum. Results obtained using this method are usually recalculated to Trolox^®^ concentration and are described as “Trolox^®^ Equivalent Antioxidant Capacity” (TEAC). For chemically pure compounds, TEAC is defined as the micromolar concentration of Trolox^®^ equivalents demonstrating the same antioxidant activity as a tested compound (at 1 mmol·L^-1^concentration) [17].

*Reagent preparation*: Seven mmol·L^-1^ ABTS^•^ (m = 0.03841 g/10 mL) and 4.95 mmol·L^-1^ potassium peroxodisulphate (m = 0.01338 g/10 mL) are mixed and dissolved in ACS water. The solution is then diluted with ACS water in a 1:9 v/v ratio (10 mL is quantitatively transferred into 100 mL calibrated flask and diluted). The solution is incubated for 12 hours in the dark and the reagent is usable for seven days if stored in the dark at 4 °C.

*Measurement procedure for an automated analyzer:* A 245 µL volume of reagent is pipetted into a plastic cuvette with subsequent addition of 5 µL of sample (gallic acid, Trolox^®^). Absorbance is measured at λ = 670 nm after 1,520 seconds. For calculating the antioxidant activity, values before decrease of the absorbance (224^th^ second of measurement – A_224_) and the last measurement value (1,520^th^ second of measurement – A_1520_) are used. Resulting value is calculated in accordance with following formula: A = A_1520_-A_224_.

#### 2.1.3. Determination of antioxidant activity by the FRAP method

The FRAP method (Ferric Reducing Antioxidant Power) is based on the reduction of complexes of 2,4,6-tripyridyl-*s*-triazine (TPTZ) with ferric chloride hexahydrate (FeCl_3_·6H_2_O), which are almost colourless, and eventually slightly brownish. This chemical forms blue ferrous complexes after its reduction. The method has its limitations, especially in measurements under non-physiological pH values (3.6). In addition, this method is not able to detect slowly reactive polyphenolic compounds and thiols [16,34].

*Reagent preparation*: Solution 1: 10 mmol·L^-1^ solution of TPTZ (m = 0.07802 g/25 mL), in 40 mM of hydrochloric acid. Solution 2: 20 mM solution of ferric chloride hexahydrate (m = 0.13513 g/25 mL) in ACS water. Solution 3: 20 mM acetate buffer, pH 3.6 (weight of sodium acetate trihydrate is 0.27216 g in 100 mL ACS water, adjusted to the desired pH using HCl). These three solutions (TPTZ, FeCl_3_, acetate buffer) are mixed in a 1:1:10 ratio. Reagent can be used for seven days if stored at 4°C in the dark.

*Measurement procedure for an automated analyzer:* A 245 µL volume of reagent is pipetted into a plastic cuvette with subsequent addition of a 5 µL sample (gallic acid, Trolox^®^). Absorbance is measured for 1,520 seconds at primary λ = 578 nm and secondary at λ = 630 nm wavelengths. For calculating the antioxidant activity, the differential absorbance (primary absorbance - secondary absorbance) is used.

#### 2.1.4. Determination of antioxidant activity by the DMPD method

The compound *N,N*-dimethyl-1,4-diaminobenzene (DMPD) is converted in solution to a relatively stable and coloured radical form by the action of ferric salt. After addition of a sample containing free radicals, these are scavenged and as a result of this scavenging, the coloured solution is decolourized [46,47].

*Reagent preparation*: Solution 1: acetate buffer (0.2 mol·L^-1^, pH 5.25); 1a) 2.17 g of sodium acetate trihydrate is dissolved in 80 mL of ACS water; 1b) 300 µL of concentrated acetic acid (>99.5 %, v/v) is diluted to a volume of 20 mL with ACS water. These two solutions are mixed to reach pH = 5.5. Solution 2: 0.74 mmol·L^-1^ ferric chloride: 1 mg of FeCl_3_·6H_2_O is dissolved with ACS water to a volume of 5 mL. Solution 3: 36.7 mmol·L^-1^ DMPD: 25 mg of DMPD is dissolved in 5 mL of ACS water. This solution must be prepared at the time of use due to its low stability. These three solutions (solutions No. 1, 2 and 3) are mixed in a 20:1:1 (*v*/*v*/*v*) ratio. 

*Measurement procedure for an automated analyzer*: A 200 µL volume of reagent is pipetted into a plastic cuvette. Then, 5 µL of sample (gallic acid, Trolox^®^) is added. Absorbance is measured at λ = 510 nm for 1,520 seconds. For calculating of the antioxidant activity, values before decrease of absorbance (224^th^ second of measurement – A_224_) and last (1520^th^ second of measurement – A_1520_) are used. Resulting value is calculated in accordance with following formula: Differential absorbance A = A_1520_ – A_224_.

#### 2.1.5. Determination of antioxidant activity by the Free Radicals method

This method is based on ability of chlorophyllin (the sodium-copper salt of chlorophyll) to accept and donate electrons with a stable change of maximum absorption. This effect is conditioned by an alkaline environment and the addition of catalyst [48]. 

*Reagent preparation*: 5 mL of reaction buffer (100 mmol·L^-1^ HCl) is diluted with 45 mL of ACS water. To this solution, 100 µL of chlorophyllin is added. After its complete dissolution, 0.25 mL of catalyst is added. Reaction buffer is stable for one month when stored at 2 - 8 °C in the dark.

*Measurement procedure for an automated analyzer:* Into a plastic cuvette, a 200 µL volume of prepared reagent is pipetted. Next, 8 µL of sample (gallic acid, Trolox^®^) is added. Absorbance is measured at λ = 450 nm for 560 seconds. For calculating of the antioxidant activity, values before decrease of the absorbance (224^th^ second of measurement – A_224_) and the last value of measurement (560^th^ second of measurement – A_560_) are used. The resulting value is calculated in accordance with the following formula: A = A_560_-A_224_.

#### 2.1.6. Determination of antioxidant activity by the Blue CrO_5_method 

Chromium peroxide (CrO_5_) is very strong pro-oxidant produced in an acidic environment by ammonium dichromate in the presence of H_2_O_2_. It is a deep blue potent oxidant compound, miscible and relatively stable in polar organic solvents that can be easily measured by spectrometry [49,50].

*Reagent preparation:* Solution 1: 1a) 10 mL of solution of sulphuric acid (25 mmol·L^-1^; 13.4 µL of 98.8 % sulphuric acid diluted with ACS water to a volume of 10 mL); 1b) 10 mL of 20 mmol·L^-1^ ammonium dichromate solution (50.4 mg of ammonium dichromate dissolved in ACS water); 1c) 30 mL of 99.5 % DMSO (*v*/*v*). These three solutions are mixed in a 1:1:3 ratio (*v*/*v*/*v*). Solution 2: 1.6 mol·L^-1^ solution of hydrogen peroxide.

*Measurement procedure for an automated analyzer:* 400 µL of solution 1 is mixed with 4 µL of sample (gallic acid, Trolox^®^, δ-tocopherol). This solution is incubated for 192 seconds. The first absorbance is measured at the 416^th^ second (A_416_). Subsequently, 40 µL of solution 2 is added and the solution is incubated for 192 seconds with measurement of the second absorbance (A_608_). Measurements are carried out at λ = 546 nm. Resulting absorbance was calculated in accordance with the formula A = A_608_ – A_416_. 

### 2.2. Analytical evaluation

Our experimental work was focused on the determination of limitations, time courses of reactions, including reaction kinetics, of the six different automated spectrometric tests - DPPH, TEAC, FRAP, DMPD, Free Radicals and Blue CrO_5_. The entire process of automatic detection proceeds as follows: a dosing needle pipettes the reagents into the cell heated to 37 °C at time T_0_. This cycle lasts 224 seconds. Subsequently, the sample is pipetted into the cell and there is a change in absorbance. In addition to other methods, at Blue CrO5 method reagents are also pipetted in the 416^th^ second.

For manually measuring of courses of spectral curves, from which their absorbance and the most suitable wavelengths for the measurement of the antioxidant activity for individual methods were determined, a SPECORD 210 spectrophotometer was used. For all methods, time courses of reactions and their reaction kinetics were determined. 

In the case of measurement of the spectra, eight concentrations of gallic acid (1; 5; 10; 50; 100; 250; 500, and 1,000 µg·mL^-1^) and eight concentrations of Trolox^®^ (10; 20; 50; 100; 250; 500; 750, and 1,000 µmol·L^-1^) were used. For measuring the calibration curves, the following concentrations of gallic acid – 1; 2; 3; 4; 5; 6; 7; 8; 9; 10; 12.5; 15; 17.5; 20; 25; 30; 40; 50; 60; 70; 80; 90; 100; 125; 150; 175; 200; 250; 300; 350; 400; 450; 500; 750 and 1,000 µg·mL^-1^ and Trolox^®^ concentrations of 10; 20; 30; 40; 50; 80; 100; 150; 200; 250; 300; 350; 400; 450; 500; 550; 600; 650; 700; 750; 800; 850; 900; 950 and 1,000 µmol·L^-1^ were used. The calibration curves were measured at constant wave- lengths, which were determined on the basis of the spectral courses. Moreover, time courses of the reaction and reaction kinetics were measured at all abovementioned concentrations of both standards. Concentrations of 1, 100 and 1 000 µg·mL^-1^ for gallic acid and concentrations 10, 100 and 1,000 µmol·l^-1^ for Trolox^®^ are shown in the figures below.

#### 2.2.1. Analytical evaluation of the DPPH^•^ test

**Figure 1:**
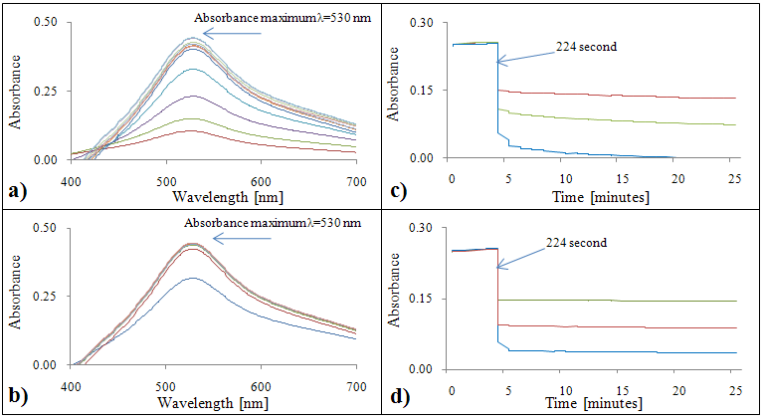
DPPH absorption spectra (400 – 700 nm) for gallic acid **(a)** and Trolox^®^ **(b)**. Dependence of absorbance of DPPH radical with gallic acid **(c)** and Trolox^®^ **(d)** on time.

Figure 1a,b show absorption maximum of the DPPH test, which was λ = 530 nm for both standards. Due to limitation of the automated analyzer, all samples were measured at λ = 510 nm. Time courses of DPPH test measured using automated analyzer are shown in Figure 1c,d. Firstly, DPPH reagent was pipetted into a cuvette. In the 224^th^ second, gallic acid/Trolox^®^ was added. In the case of gallic acid, a slight reduction of absorbance was observed with increasing time (Figure 1c). Therefore, it is necessary to analyze samples within 1,592 seconds. For Trolox^®^, the absorbance was constant (Figure 1d).

#### 2.2.2. Analytical evaluation of the ABTS test

**Figure 2:**
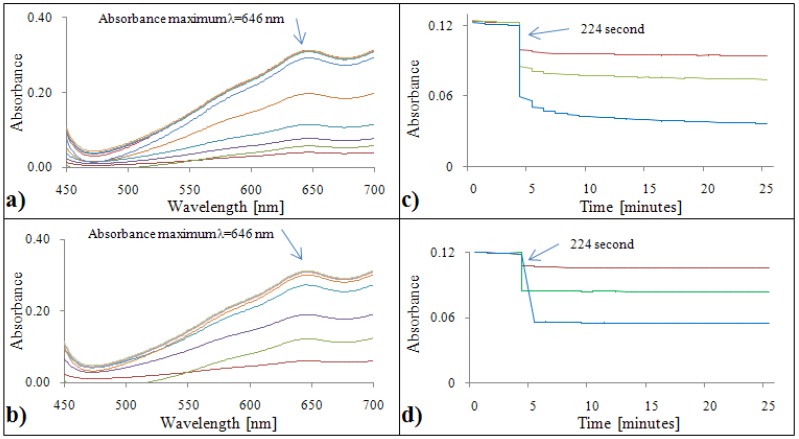
ABTS absorption spectra (450 – 700 nm) for gallic acid **(a)** and Trolox^®^ **(b)**. Dependence of absorbance of ABTS radical with gallic acid **(c)** and Trolox^®^ **(d)** on time.

Figure 2a,b show the absorption maximum of the ABTS test, which was λ = 646 nm for both standards. Due to limitations of the automated analyzer, all samples were measured at an absorbance λ = 670 nm. Time courses of ABTS test reactions measured using the automated analyzer are shown in Figure 2c,d. The procedure was identical to the DPPH test. Firstly, the reaction reagent ABTS was pipetted into the cuvette. In the 224^th^ second, gallic acid/Trolox^®^ was added. In the case of gallic acid, a slight decrease of absorbance was observed with increasing time (Figure 2c). In the case of the Trolox^®^ standard the absorbance was constant from the 224^th^ to the 1592^th^ second (Figure 2d).

#### 2.2.3. Analytical evaluation of the FRAP method

**Figure 3:**
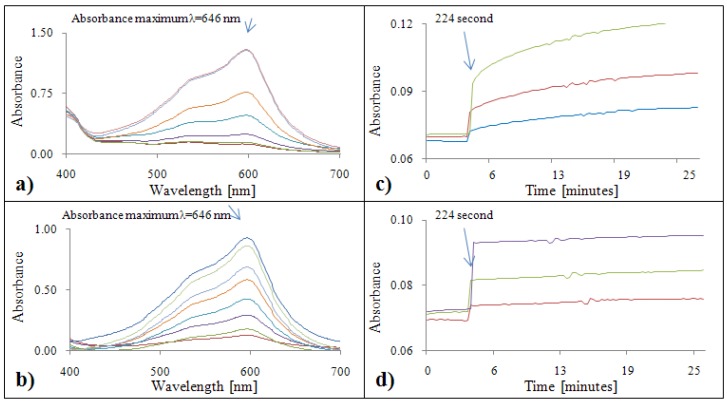
FRAP absorption spectra (450 – 700 nm) for gallic acid **(a)** and Trolox^®^ **(b)**. Dependence of absorbance of FRAP with gallic acid **(c)** and Trolox^®^ **(d)** on time.

Figure 3a,b show absorption maximum of the FRAP method, which was λ = 596 nm for both standards. To obtain more accurate data, measurements at two different wavelengths, primary absorbance λ = 578 nm and secondary absorbance λ = 630 nm, were carried out. The absorbance of gallic acid and FRAP reagent was enhanced with increasing time (Figure 3c). FRAP reagent with Trolox^®^ also demonstrated a clearly evident increasing tendency. Based on the results obtained, the absorbance measured in the 1520^th^ second was used for the subsequent calculation (Figure 3d).

#### 2.2.4. Analytical evaluation of the DMPD method

**Figure 4:**
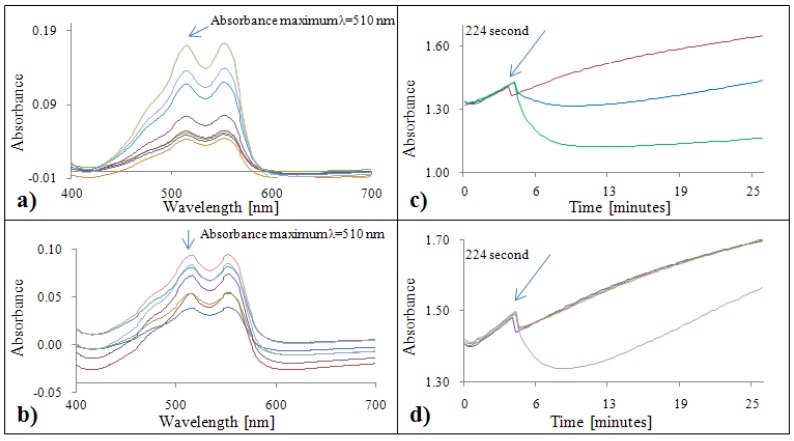
DMPD absorption spectra (450 – 700 nm) for gallic acid **(a)** and Trolox^®^ **(b)**. Dependence of absorbance of DMPD with gallic acid **(c)** and Trolox^®^ **(d)** on time.

Based on the courses of the DMPD absorption spectra with gallic acid (Figure 4a) and Trolox^®^ (Figure 4b), it is clearly evident that the highest absorbance can be measured at two wavelengths: λ = 515 nm and λ = 553 nm. With respect to limitations of the automated analyzer, we chose λ = 510 nm for our subsequent experiments. A decrease in the absorbance was observed with increasing antioxidant activity expressed as increasing concentration of gallic acid as well as Trolox^®^. The reaction kinetics for gallic acid and DMPD radical were very dynamic during 1,592 seconds (Figure 4c). In the case of low concentrations, an increase in the absorbance was observed (to a concentration of 5 µg·mL^-1^) . With increasing concentration of Trolox^®^, the absorbance decreased to the 560^th^ second and increased from the 576^th^ second (Figure 4d).

#### 2.2.5. Analytical evaluation of the Free Radicals method

**Figure 5:**
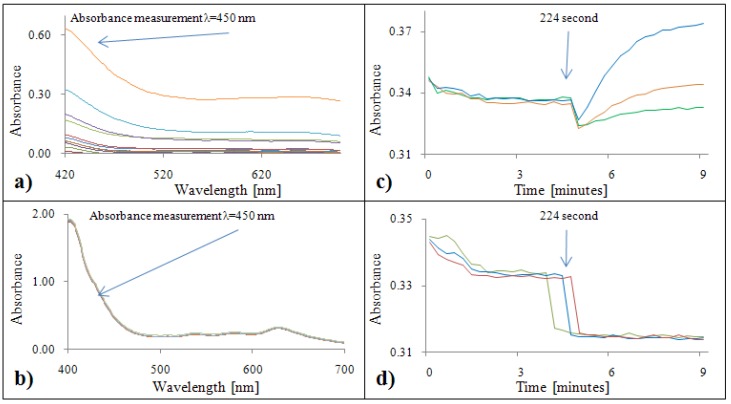
Free Radicals absoprtion spectra (450 – 700 nm) for gallic acid **(a)** and Trolox^®^ **(b)**. Dependence of absorbance of Free Radicals with gallic acid **(c)** and Trolox^®^ **(d)** on time.

The course of the Free Radicals absorption spectrum with Trolox^®^ did not change with the increasing concentration of Trolox^®^ (Figure 5b). This phenomenon was verified also by the time course of the reaction (Figure 5d), where all studied concentrations demonstrated relatively the same or very similar absorbance values. The absorbance enhanced linearly with the increasing wavelength for gallic acid. Its increasing tendency is also well evident in curves demonstrating the time dependence. The absorption maximum was 510 nm, which was also suitable for setting our automated analyzer, so all values were obtained by measuring at 510 nm. At the 512^th^ second the absorbance was constant, thus, this absorbance was used for the following calculation (Figure 5c).

#### 2.2.6. Analytical evaluation of the Blue CrO_5_ method

**Figure 6:**
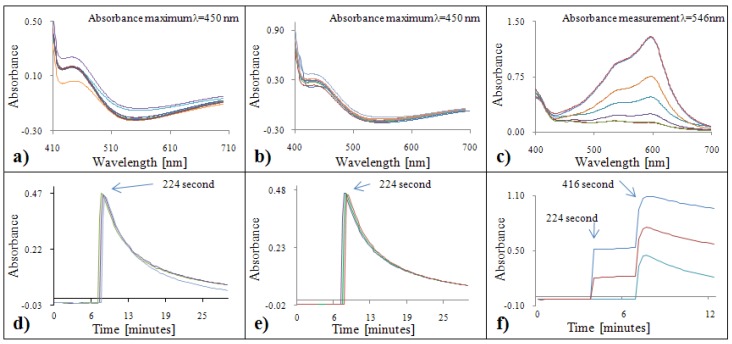
Blue CrO_5_ absorption spectra (450 – 700 nm) for gallic acid **(a)**, Trolox^®^ **(b)** and δ tocopherol **(c)**. Dependence of absorbance of Blue CrO_5_ with gallic acid **(c)**, Trolox^®^ **(d)** and δ tocopherol **(c)** on time.

It is well evident from the absorption spectra (Figure 6a,b) as well as from time courses of the reactions (Figure 6d,e) that absorbences were practically constant at various concentrations of Trolox^®^ and gallic acid. This fact is caused by inability of gallic acid and Trolox^®^ to react with the oxidant CrO_5_. Due to this fact, we used δ-tocopherol in this experiment (Figure 6c,f). The absorption maximum was measured at 608 nm. Due to limitations of the automatic analyser, a wavelength of 546 nm was used for the subsequent experiments. The absorbance time course demonstrates its great increase after addition of reagents (solution of sulphuric acid, solution of ammonium dichromate and DMSO). After a 416 second long incubation, hydrogen peroxide was added into the cuvette. Subsequently, a decrease of absorbance was determined. After a 192 seconds long incubation, the absorbance of reagents and tocopherol did not change. Values measured in 608^th^ second were used for the calculation of the antioxidant activity.

### 2.3. Calibration

All methods were calibrated on both the automated analyzer (BS-200) and a two beam UV-VIS spectrophotometer SPECORD 210 on standards of the antioxidants - Trolox^®^ and gallic acid (the blue CrO_5_ method was also calibrated on δ-tocopherol). Further, calibration curves were measured. In an effort to achieve reliable data, all measurement methods were repeated five times. Average values were calculated from these data. The most important aspect was the duration of the analysis itself. Time of analysis ranged from 10 minutes (Blue CrO_5_ method) to 25 minutes (DPPH test) in the case of use of the manual spectrophotometer. Including the time needed for pipetting of the individual samples, manual manipulation with cuvettes, operation of the apparatus and evaluation of data, the time of measurement for one sample varied from 15 to 30 minutes. In the case where all six methods are used, measurement of one sample takes 120 – 150 minutes. Using an automated analyzer, 40 samples can be measured in one run. In addition, manipulation with samples, including pipetting and recalculation of the measured data (methods are automatically calibrated), is fully automated. In this case, the time of analysis of a set of samples (40) varies from 35 minutes (Blue CrO_5_ method) to 80 minutes (DPPH test), which means from 1 to 2 minutes per sample. For all six methods used, analysis of one sample takes 10 minutes. Due to automation, errors incoming from handling of a sample and consumption of all chemicals are reduced. 

#### 2.3.1. Calibration of the DPPH^•^ test

**Figure 7:**
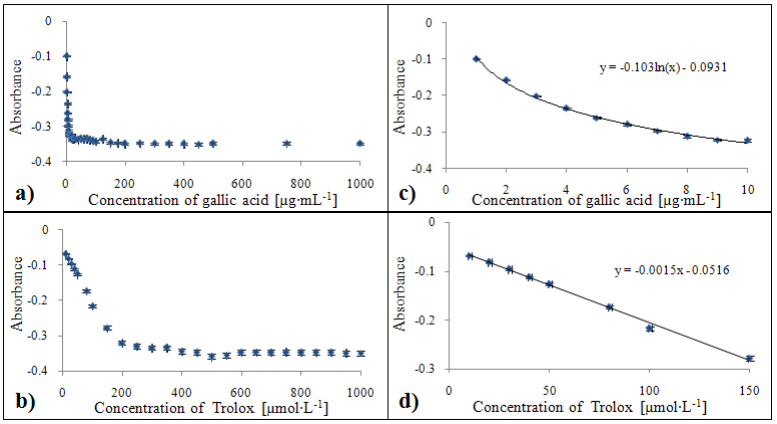
Dependences of absorbance of colour product on concentration of gallic acid **(a)** and Trolox^®^ **(b)**. Calibration curves for DPPH test related to equivalent of gallic acid **(c)** and Trolox^®^ **(d)** expressed as the percentage of inhibition.

Dependences of the rising signal intensity of coloured product on concentration of gallic acid (a) and Trolox^®^ (b) related to percentage of inhibition are shown in Figure 7a,b. The method of determination of the antioxidant activity based on the DPPH^•^ test can only be used for samples with low antioxidant activity values (gallic acid: 1 – 10 µg·mL^-1^, Trolox^®^: 10 – 150 µmol·L^-1^). In the case of higher concentrations, the absorbance did not change. Due to this fact, it is necessary to dilute analysed samples to the measurable range mentioned above. The calibration curve equation related to standard of gallic acid was y = -0.103 ln(x) - 0.0931 with confidence coefficient R^2^ = 0.996 and relative standard deviation 1.8 % within the concentration range from 1 to 10 µg·mL^-1^ (Figure 7c). For the Trolox^®^ standard, this equation was determined as y = -0.0015x - 0.0516 with R^2^ = 0.996 with relative standard deviation 2.4 % related to Trolox^®^ concentration within a concentration range from 10 to 150 µmol·L^-1^ (Figure 7d).

#### 2.3.2. Calibration of the ABTS method

**Figure 8:**
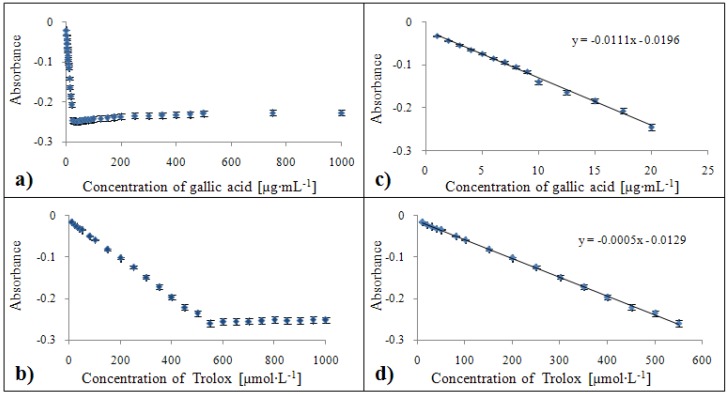
Dependence of absorbance of colour product on concentration of gallic acid **(a)** and Trolox^®^ **(b)**. Calibration curves for ABTS method related to equivalent of gallic acid **(c)** and Trolox^®^ **(d)** expressed as percentage of inhibition.

Changes of absorbance expressed as percentage of inhibition for individual concentrations of gallic acid (a) and Trolox^®^ (b) are shown in Figure 8a,b. Based on the calibration by gallic acid, ABTS radical can be used for determination of the antioxidant activity up to 20 µg·mL^-1^ and in the case of Trolox^®^ up to the 550 mmol·L^-1^. At higher concentrations, the percentage of inhibition remained constant, which makes determination of higher concentrations practically impossible. The calibration curve equation related to the gallic acid standard was y = -0.0111x - 0.0196 with a confidence coefficient R^2^ = 0.996 and a relative standard deviation of 2.1 % within a concentration range from 1 to 20 µg·mL^-1^ (Figure 8a). For the Trolox^®^ standard, this equation was determined as y = -0.0005x - 0.0129 with R^2^ = 0.999 with a relative standard deviation of 1.5 % within a concentration range from 10 to 550 µmol·L^-1^ (Figure 8b). 

#### 2.3.3. Calibration of the FRAP method

**Figure 9:**
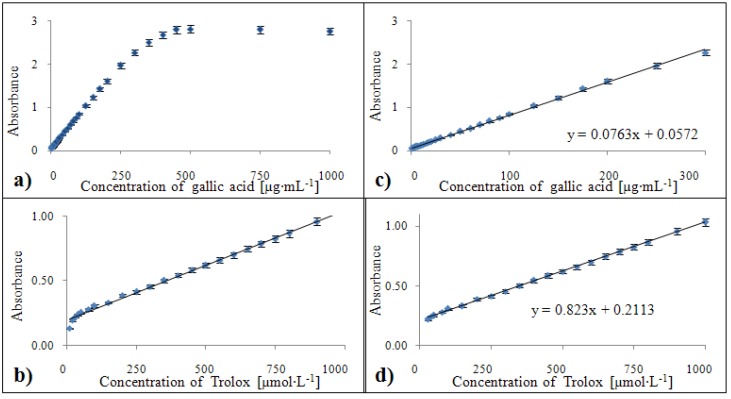
Dependences of absorbance of colour product on concentration of gallic acid **(a)** and Trolox^®^ **(b)**. Calibration curves for FRAP method related to equivalent of gallic acid **(c)** and Trolox^®^ **(d)** expressed in units of absorbance.

This method has limitations for gallic acid in concentrations higher than 300 µg·mL^-1^ (Figure 9a). Calibration on a Trolox^®^ standard showed that linearity was only not reached for the two lowest values and the bottom limit for the calibration curve was 30 µmol·L^-1^ (Figure 9b). The calibration curve equation related to the gallic acid standard was y = 0.0763x + 0.0572 with a confidence coefficient R^2^ = 0.999 and relative standard deviation 1.5 % within the 1 – 300 µg·mL^-1^ concentration range (Figure 9c). For the Trolox^®^ standard, this equation was determined as y = 0.8286x + 0.2101 with R^2^ = 0.996 and relative standard deviation 1.5 % within the concentration range from 30 to 1,000 µmol·L^-1^ (Figure 9d).

#### 2.3.4. Calibration of the DMPD method

**Figure 10:**
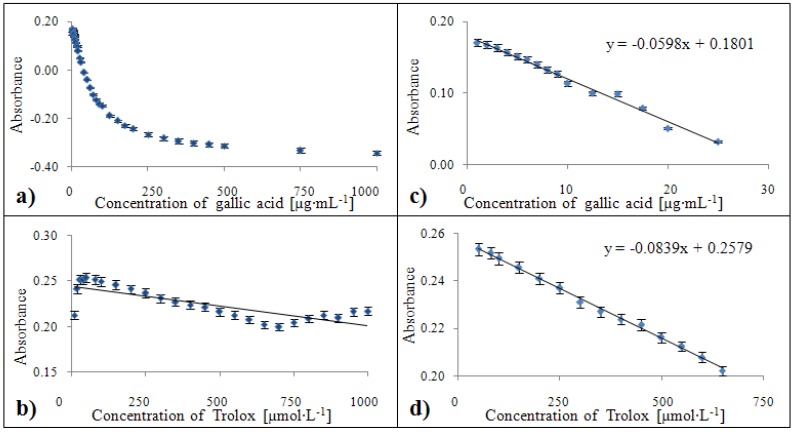
Dependences of absorbance of colour product on concentration of gallic acid (**a**) and Trolox^®^ (**b**). Calibration curves for DMPD method related to equivalent of gallic acid (**c**) and Trolox^®^ (**d**) expressed in units of absorbance.

The absorbances showed a decreasing tendency for gallic acid at concentrations higher than 25 µg·mL^-1^ (Figure 10a). The calibration curve equation of the gallic acid standard was y = -0.0598x + 0.1801 with a confidence coefficient R^2^ = 0.999 and relative standard deviation 2.3 % for concentrations within the 1 – 25 µg·mL^-1^ range (Figure 10c). For the standard of Trolox^®^, this dependence was determined by the equation y = -0.0839x + 0.2579 with R^2^ = 0.997 with a relative standard deviation of 1.9 % within a concentration range from 50 to 650 µmol·L^-1^ (Figure 10d).

#### 2.3.5. Calibration of the Free Radical method

**Figure 11:**
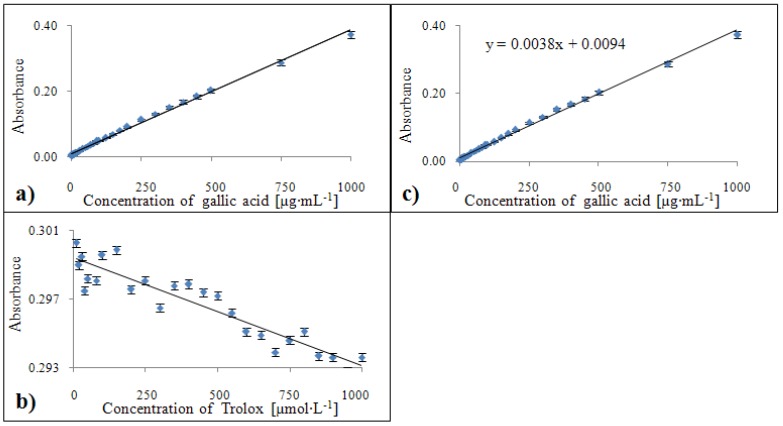
Dependences of absorbance of colour product on concentration of gallic acid **(a)** and Trolox^®^ **(b)**. Calibration curve for the Free radical method related to equivalents of gallic acid **(c)**, expressed in units of absorbance.

The Trolox^®^ standard is not suitable for calibration of the Free Radical method (Figure 11b). The concentration of gallic acid on the other hand correlated with absorbance for all concentrations used except 1 µg·mL^-1^ (Figure 11a). The calibration curve equation for the gallic acid standard was y = 0.035x + 0.2667 with a confidence coefficient R^2^ = 0.996 and relative standard deviation 1.5 % (Figure 11c).

#### 2.3.6. Calibration of the Blue CrO_5_ method

**Figure 12:**
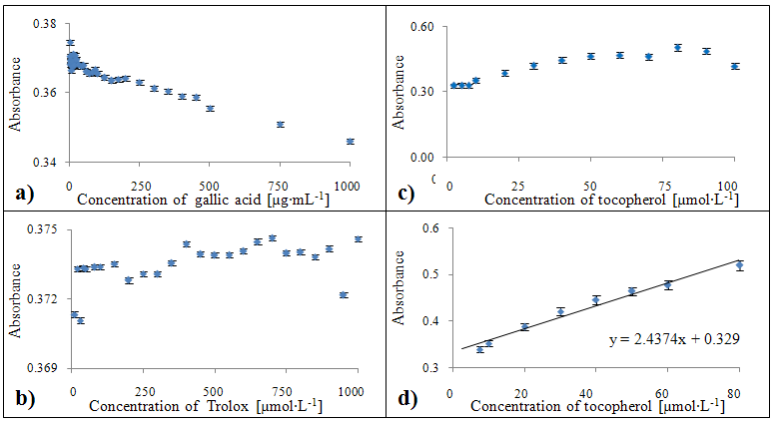
Dependences of absorbance of colour product on concentration of gallic acid **(a)**, trolox **(b)** and tocopherol **(c)**. Calibration curves for Blue CrO_5_ method related to equivalent of tocopherol **(d)** expressed in units of absorbance.

In the case of the Blue CrO_5_ method, it was not possible to establish a relevant calibration dependence for gallic acid and Trolox^®^ (see Figure 12a,b), so δ-tocopherol was chosen as the most suitable standard for comparing antioxidant activity (Figure 12c) [43,44]. A calibration curve was obtained for the concentration range from 5 to 80 µmol·L^-1^ of tocopherol. The calibration curve obtained (Figure 12d) had the following parameters: y = 2.5396x + 0.330 with a confidence coefficient R^2^ = 0.973 and relative standard deviation 2.5 % .

## 3. Experimental 

### 3.1. Apparatus

In this study, a BS-200 automated spectrophotometer (Mindray, China) was used. It is composed of cuvette space tempered to 37±1 °C, reagent space with a carousel for reagents and preparation of samples (tempered to 4±1 °C) and an optical detector. Transfer of samples and reagents is provided by robotic arm equipped with a dosing needle (error of dosage up to 5 % of volume). Cuvette contents are mixed by an automatic mixer including a stirrer immediately after addition of reagents or samples. Contamination is reduced due to its rinsing system, including rinsing of the dosing needle as well as the stirrer by MilliQ water. For detection itself, the following range of wave lengths can be used - 340, 405, 450, 510, 546, 578, 630, and 670 nm. In addition, a Specord 210 two beam UV-VIS spectrophotometer (Chromspec, Czech Republic) with cooled semiconductor detector for measurement within range from 190 to 1,100 nm with control by an external PC with the programme WinASPECT was used as the manual instrument in this study. Laboratory scales (Sartorius, Germany) and pipettes (Eppendorf Research, Germany) were used.

### 3.2. Chemicals

Trolox^®^—A water soluble derivative of vitamin E (6-hydroxy-2,5,7,8-tetramethylchroman-2-carboxylic acid), standard of gallic acid, free DPPH^•^ radical (2,2-diphenyl-1-picrylhydrazyl), dimethyl sulfoxide (DMSO), the synthetic chromophore ABTS^•^ (2,2‘-azino-bis(3-ethylbenzothiazoline-6-sulfonic acid)), potassium peroxodisulfate, TPTZ (2,4,6-tripyridyl-*s*-triazine), ferric chloride hexahydrate, sodium acetate trihydrate, hydrochloric acid, sulphuric acid, DMPD (*N,N*-dimethyl-1,4-diaminobenzene), ammonium dichromate, hydrogen peroxide, δ-tocopherol, ACS water, 99% methanol (*v*/*v*) were purchased from Sigma Aldrich (St. Louis, MO, USA). Reaction buffer, chlorophyllin concentrate and its catalyst were purchased from Sedium R&D (Czech Republic).

### 3.3. Standards

As standards, Trolox^®^ in 25 different concentrations - 10; 20; 30; 40; 50; 80; 100; 150; 200; 250; 300; 350; 400; 450; 500; 550; 600; 650; 700; 750; 800; 850; 900; 950, and 1,000 µmol·L^-1^ and gallic acid in 35 different concentrations *-* 1; 2; 3; 4; 5; 6; 7; 8; 9; 10; 12; 15; 17; 20; 25; 30; 40; 50; 60; 70; 80; 90; 100; 125; 150; 175; 200; 250; 300; 350; 400; 450; 500; 750, and 1,000 µg·mL^-1^ were used. For the blue CrO_5_ method, δ-tocopherol in concentrations 2; 5; 7.5; 10; 20; 30; 40; 50; 60; 70; 80; 90; and 100 mmol·L^-1^ was prepared. Stock solutions of Trolox^®^ was prepared by diluting Trolox^®^ with 99% methanol (*v*/*v*) in a concentration of 10 mmol·L^-1^. Working standard solutions were prepared daily by dilution of the stock solutions with ACS water to the concentration of 1 mmol·L^-1^ and lower. δ-Tocopherol was diluted with 99% methanol to a concentration of 1 mmol·L^-1^ and subsequently diluted to the final, abovementioned concentrations as needed. Stability of Trolox^®^ standard solution is 24 hours at 4 °C. Stock solution of gallic acid is stable for 72 hours at 4 °C.

### 3.4. UV-Vis spectrometric protocols

#### 3.4.1. DPPH

A volume of 1,000 µL of reagent was pipetted into plastic cuvettes. Subsequently, a volume of 100 µL of the standard (gallic acid, Trolox^®^) was added. Absorbance was measured at λ = 510 nm. For calculation value of absorbance of reagent itself (A_R_) and value determined after 25 minutes of incubation A_25_ was used. Resulting value was calculated in accordance with following formula: A = A_25_-A_R._

#### 3.4.2. ABTS

A volume of 980 µL of reagent and subsequently a volume of 20 µL of measured sample (gallic acid, Trolox^®^) was added into plastic cuvettes. Absorbance was measured at λ = 670 nm. For calculation of the absorbance of the reagent itself (A_R_) and value determined after 25 minutes of incubation A_25_ was used. Resulting value was calculated in accordance with following formula: A = A_25_-A_R_

#### 3.4.3. FRAP

A volume of 980 µL of reagent and subsequently a volume of 20 µL of measured sample (gallic acid, Trolox^®^) was pipetted into plastic cuvettes. Absorbance was measured after 25 minutes of incubation at primary λ = 578 nm and secondary λ = 630 nm wave lengths. The obtained values of absorbance were subtracted (primary absorbance - secondary absorbance = differential absorbance). For calculating of antioxidant activity, differential absorbance was used. 

#### 3.4.4. DMPB 

A volume of 1,000 µL of reagent was added into plastic cuvettes with subsequent addition of 25 µL of sample (gallic acid, Trolox^®^). Absorbance was measured at λ = 510 nm. For calculation of the absorbance value of the reagent itself (A_R_) and value of absorbance determined after 25 minutes of incubation (A_25_) was used. Resulting value was calculated in accordance with following formula: differential absorbance = A_25_ – A_R_.

#### 3.4.5. Free radicals

A volume of 1,000 µL of prepared reagent was pipetted into plastic cuvettes with immediate addition of 40 µL of sample (gallic acid, Trolox^®^). Absorbance was measured after 9 minutes at λ = 450 nm. For calculation of the absorbance value of the reagent itself (A_R_) the absorbance determined after 25 minutes of incubation (A_25_) was used. Resulting value was calculated in accordance with following formula: A = A_25_-A_R._

#### 3.4.6. Blue CrO_5_

Solution No. 1 (1,200 µL) was pipetted into plastic cuvettes and mixed with sample solution (12 µL, gallic acid, Trolox^®^, δ-tocopherol). Incubation took 3 minutes. After that, the first value of absorbance (A_1_) was determined. Subsequently, a volume of 120 µL of reagent No. 2 was added and incubated for 3 minutes. After this incubation, a second absorbance value was determined at λ = 546 nm (A_2_). Resulting absorbance was calculated according to the formula A = A_2_ - A_1_.

### 3.5. Descriptive statistics

Data were processed using MICROSOFT EXCEL® (USA) and STATISTICA.CZ Version 8.0 (Czech Republic). Results are expressed as mean ± standard deviation (S.D.) unless noted otherwise (EXCEL®).

## 4. Conclusions

The aim of this study was to find and describe the limitations, time courses of reactions, and reaction kinetics of six different photometric protocols. The study resulted in the optimization, automation and precise description of protocols of six photometric methods, namely the DPPH, TEAC, FRAP, DMPD, Free Radicals Kit and Blue CrO_5_ assays, which cand be used for the determination of antioxidant activity. 

On the basis of our measurements of standard compounds—Trolox^®^, gallic acid and tocopherol—calibration curves with high confidence coefficients (r^2^ = 0.97 – 0.99) were determined (Table 1, Table 2 and Table 3). In addition, standard deviations were very low, within the 1.50 to 2.50 % Range. Each technique is based on different principles and enables determination of the antioxidant activity of specific groups of compounds. These techniques have their limitations, thus, it is necessary to determine antioxidant activity using different techniques with appropriate dilution of analysed samples.

Time of analysis is also a very important parameter. In the case of fully automated analysis, the duration of analysis varied from 1 to 2 min per sample (including pipetting), in comparison with a manual measurement, at which duration of a measurement varied from 20 to 40 min per sample. Due to the automation of measurements, numerous operation and handling errors are eliminated. In addition, consumption of all chemicals used is reduced. In the case of fully automated analyses, it is possible to analyze more samples in one run.

## Figures and Tables

**Table 1:** Summarization of the parameters for individual methods related to the standard of gallic acid (n = 5).

Method	Wavelength [nm]	Interaction Time [sec.]	Measuring range [µg·mL^-1^]	Calibration equation	Confidence coefficient [R^2^]	Standard deviation [%]
DPPH	510	1296	1 – 10	y = -0.103ln(x) - 0.0931	0.996	1.80
ABTS	670	1296	1 – 20	y = -0.0111x - 0.0196	0.996	2.10
FRAP	578/630	1296	1 – 300	y = 0.076x + 0.057	0.999	1.50
DMPD	510	1296	1 – 25	y = -0.060x + 0.180	0.999	2.30
FR	450	560	1 – 1000	y = 0.035x + 0.267	0.996	1.50

**Table 2:** Summarization of the parameters for individual methods related to the standard of Trolox^®^ (n = 5).

Method	Wavelength [nm]	Interaction Time [sec.]	Measuring range [µmol·L^-1^]	Calibration equation	Confidence coefficient [R^2^]	Standard deviation [%]
DPPH	510	1296	10 – 150	y = -0.0015x - 0.0516	0.996	2.40
ABTS	670	1296	10 – 550	y = -0.0005x - 0.0129	0.999	1.50
FRAP	578/630	1296	30 – 1000	y = 0.829x + 0.210	0.996	1.50
DMPD	510	1296	50 – 650	y = -0.084x + 0.257	0.997	1.90
FR	450	560	—	—	—	—

**Table 3:** Summarization of the parameters for individual methods related to standard of δ- tocopherol (n = 5).

Method	Wavelength [nm]	Interaction Time [sec.]	Measuring range [µmol·L^-1^]	Calibration equation	Confidence coefficient [R^2^]	Standard deviation [%]
CrO_5_	546	224	5 - 80	y = 2.530x + 0.3300	0.973	2.50

## Abbreviations

ABTS – 2,2‘-azino-bis(3-ethylbenzothiazoline-6-sulfonic acid); ACS – chemicals meeting the specifications of the American Chemical Society; AH – antioxidant; CrO5 - chromium peroxide; DMPD – *N,N*-dimethyl-1,4-diaminobenzene; DMSO – dimethyl sulfoxide; DPPH – 2,2-diphenyl-1-picrylhydrazyl; FeCl_3_ – ferric chloride; FR – free radical; FRAP – Ferric Reducing Antioxidant Power; HCl – hydrochloric acid; OS – oxidative stress; PC – personal computer; R• – radical; TEAC – Trolox^®^ Equivalent Antioxidant Capacity; TPTZ – 2,4,6-tripyridyl-*s*-triazine; Trolox^®^ – 6-hydroxy-2,5,7,8-tetramethylchroman-2-carboxylic acid; UV-VIS – ultraviolet - visible spectrum.
